# The Arrival of Gene Therapy for Patients with Hemophilia A

**DOI:** 10.3390/ijms231810228

**Published:** 2022-09-06

**Authors:** Giancarlo Castaman, Giovanni Di Minno, Raimondo De Cristofaro, Flora Peyvandi

**Affiliations:** 1 Center for Bleeding Disorders, Department of Oncology, Careggi University Hospital, Largo Brambilla 3, 50134 Firenze, Italy; 2 Regional Reference Centre for Hemo-Coagulation Diseases, Federico II University, Via S. Pansini 5, 80131 Naples, Italy; 3 Servizio Malattie Emorragiche e Trombotiche, Dipartimento di Medicina e Chirurgia Traslazionale, Fondazione Policlinico Universitraio “A. Gemelli” IRCCS, Università Cattolica S. Cuore Roma, Largo Francesco Vito, 1, 00168 Rome, Italy; 4 Angelo Bianchi Bonomi Hemophilia and Thrombosis Center, Fondazione Luigi Villa, Fondazione IRCCS Ca’ Granda Ospedale Maggiore Policlinico, 20122 Milan, Italy; 5 Department of Pathophysiology and Transplantation, Università degli Studi di Milano, Via Pace 9, 20122 Milan, Italy

**Keywords:** adeno-associated viral vector, emicizumab, exogenous factor VIII, gene therapy, hemophilia A, valoctogene roxaparvovec

## Abstract

Historically, the standard of care for hemophilia A has been intravenous administration of exogenous factor VIII (FVIII), either as prophylaxis or episodically. The development of emicizumab, a humanized bispecific monoclonal antibody mimicking activated FVIII, was a subsequent advance in treatment. However, both exogenous FVIII and emicizumab require repeated and lifelong administration, negatively impacting patient quality of life. A recent breakthrough has been the development of gene therapy. This allows a single intravenous treatment that could result in long-term expression of FVIII, maintenance of steady-state plasma concentrations, and minimization (or possibly elimination) of bleeding episodes for the recipient’s lifetime. Several gene therapies have been assessed in clinical trials, with positive outcomes. Valoctocogene roxaparvovec (an adeno-associated viral 5-based therapy encoding human B domain-deleted FVIII) is expected to be the first approved gene therapy in European countries, including Italy, in 2022. Some novel challenges exist including refining patient selection criteria, managing patient expectations, further elucidation of the durability and variability of transgene expression and long-term safety, and the development of standardized ‘hub and spoke’ centers to optimize and monitor this innovative treatment. Gene therapy represents a paradigm shift, and may become a new reference standard for treating patients with hemophilia A.

## 1. Introduction

Hemophilia is a hemorrhagic disease that includes two distinct genetic disorders caused by missing or defective clotting factor VIII (FVIII; hemophilia A) or clotting factor IX (hemophilia B) [1,2,3,4,5,6].

FVIII is a large protein that is endogenously produced, primarily in the liver by sinusoidal endothelial cells (rather than hepatocytes) [4,7,8,9]. Disease severity in hemophilia A is classified according to the plasma level of FVIII activity [2,10]. The severe form of hemophilia A is defined as a FVIII activity level <1% of normal, the moderate form as a FVIII level of 1–5% of normal, and the mild form as a FVIII level >5% to <40% of normal [10]. Endogenous FVIII activity levels of <1 IU/dL (1%) present an increased risk of spontaneous and trauma-related bleeding events, including bleeding into the joints that leads to hemophilic arthropathy. In contrast, an endogenous FVIII activity level >1 IU/dL is associated with a low risk of breakthrough bleeding and progressive joint destruction [11,12].

Currently available therapeutic options for hemophilia A (primarily, intravenous administration of exogenous FVIII) are associated with several limitations and there is an unmet need for improved care in these patients. Gene therapy represents an emerging effective long-term treatment modality for hemophilia that potentially bypasses the complications of other therapies, reducing the number of breakthrough bleeding events and the need for frequent infusions, thereby improving patient health-related quality of life (QoL) [4]. Despite its potential, several issues remain to be fully clarified for hemophilia A gene therapy, including patient selection criteria, the durability and variability of transgene expression, and long-term safety. In addition, the complexity and potential complications of prescribing, administering, and monitoring patients undergoing gene therapy necessitate specific considerations for the centers that will offer this therapy, i.e., expert ‘hubs’, in which a multidisciplinary team will be required to ensure maximum benefit from therapy and optimal levels of treatment and care.

This paper provides an overview of the results of clinical trials of gene therapy for hemophilia A, outlines challenges and opportunities for patients, and provides an expert opinion on the arrival in Italy of gene therapy for patients with hemophilia A.

## 2. Currently Available Therapies and Their Limitations

### 2.1. Exogenous Coagulation Factors

#### Factor VIII

Currently, intravenous administration of exogenous FVIII, either as prophylaxis or episodically to treat bleeding events, trauma, or surgical procedures, constitutes the gold standard in hemophilia A treatment [2,13,14,15]. However, exogenous coagulation factors represent a significant burden to patients, impacting their health-related QoL.

The short biological half-life of the standard FVIII protein requires frequent infusions (2–3 times per week) to maintain trough levels above 1%. To overcome this burden, long-acting FVIII products have been developed that require less frequent intravenous infusions, although the half-life of these products is only 1.4–1.5-times longer than the previously available short-acting products [16]. The requirement for frequent dosing may create venous access problems, poses an obstacle to patient adherence and the proper use and adoption of prophylaxis, and is associated with significant financial costs, which have resulted in substantial global variation in access to this standard of care.

Further, prophylaxis with exogenous coagulation factors can be associated with a “sawtooth” pattern of plasma FVIII levels, in which FVIII increases to high levels immediately after infusion and then falls rapidly to near baseline, which can lead to breakthrough bleeding. In addition, prophylaxis requires significant planning in everyday life. This is particularly important for individuals participating in high-risk activities, such as contact sports.

An immune response against therapeutic FVIII can seriously complicate replacement factor treatment [2,5], due to the development of neutralizing alloantibodies (inhibitors) to FVIII that render the exogenous factor ineffective [5,17,18]. The incidence of neutralizing antibody development is the highest in patients with severe hemophilia A (up to 30%) [18,19].

Finally, prophylactic FVIII therapy is not effective for all patients, with arthropathy still developing in some patients, further reducing their QoL [20,21].

### 2.2. Emicizumab

In addition to long-acting FVIII products, another step towards improving QoL for patients with hemophilia A has been the development of the FVIII-mimicking product emicizumab.

Emicizumab is a humanized bispecific monoclonal antibody that binds, and bridges activated factor IX and factor X, mimicking the function of activated FVIII [14,22,23,24,25]. Emicizumab is administered subcutaneously and regardless of the presence or absence of inhibitors [26]. However, emicizumab must be administered as maintenance therapy (every 7, 14, or 28 days) [14,22,27], and breakthrough bleeds and surgery still require the use of FVIII or by-passing agents in patients with inhibitors.

## 3. Gene Therapy in Hemophilia A

Gene therapy is a treatment strategy used to repair or provide a functional copy of a gene that is either absent or expressed as a non-functional protein [4,28,29,30]. Treating a genetic disease with gene therapy requires that the transgene (or its protein product) be delivered to the physiologically relevant target tissue or tissues, be stably expressed, and not interfere with the functional integrity of the cells in these tissues [31,32,33]. The ultimate goal of gene therapy for patients with hemophilia A is the production of a treatment that is given as a one-time infusion and that allows adequate long-term expression of the deficient FVIII, with the maintenance of steady-state plasma FVIII concentrations. This would minimize (or ideally eliminate) bleeding episodes and thereby decrease the patient and societal burden of the disease [34,35,36,37].

After more than three decades of investigation, gene therapy in hemophilia A has moved beyond proof of concept, toward a realistic expectation that the first product may soon be available. Indeed, such a gene therapy is likely to be approved in Europe in 2022.

Hemophilia A is an ideal candidate for gene therapy [32,38,39,40,41,42]. Its well-characterized monogenic nature of inheritance allows for the correction of a single gene to provide symptomatic relief [32,43]. In hemophilia A, gene transfer strategies employing adeno-associated viral (AAV) vectors to target liver hepatocytes are the closest to regulatory approval. Lentiviral vectors (LVs) targeting hematopoietic stem and progenitor cells ex vivo have shown promise in animal models [44] and have advanced into clinical testing [6]; however, lentiviral vectors integrate into DNA and may potentially induce oncogenesis [45,46,47].

### Adeno-Associated Viral Vectors

**Figure 1:**
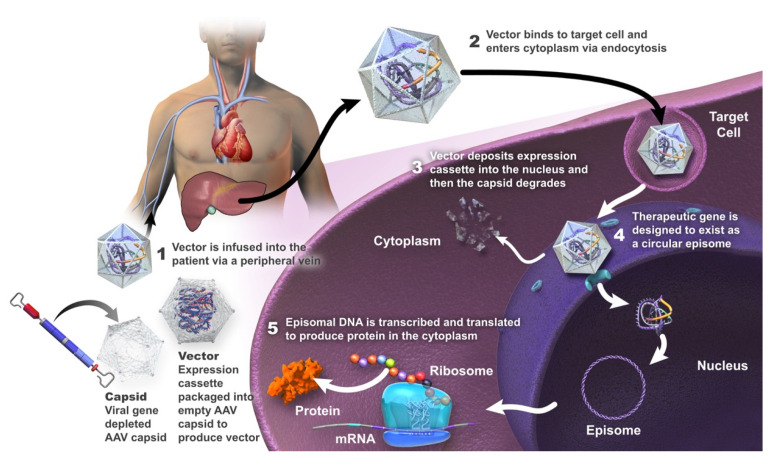
Summary of mechanism of action of gene therapy using an adeno-associated viral (AAV) vector.

To date, AAV vectors have demonstrated the greatest clinical success for in vivo gene delivery [29,48,49], and will be the focus of this article (Figure 1).

AAV vectors are engineered from a non-pathogenic, non-enveloped linear, single-stranded DNA parvovirus; wild-type AAV is naturally replication-defective and requires a helper virus for replication [4,50,51,52]. These vectors are minimally integrative and associated with a low risk of insertional mutagenesis [48,53,54,55]. The host transcription machinery transcribes the transgene into mRNA, which is then translated into the protein of interest [6]. It is considered that the genome of the recombinant AAV vector does not undergo site-specific integration in the host DNA or alter the genetic process, but is largely episomal in the nucleus of transduced cells [48].

## 4. Hemophilia A Clinical Trials Involving Gene Therapy

Several AAV-based gene therapies for hemophilia A are currently under evaluation in clinical studies (Table 1) [56,57,58,59,60,61,62,63,64,65,66,67,68,69,70,71]. In these clinical trials, FVIII replacement therapy was administered in the event of a breakthrough bleeding episode [58,62,65], as gene therapy does not preclude “rescue” therapy, or the return to standard therapy should gene therapy fail.

### 4.1. Valoctocogene Roxaparvovec

Valoctocogene roxaparvovec is a recombinant codon-optimized AAV5 vector that expresses the SQ variant of B-domain-deleted human FVIII with a hybrid liver-specific transcription promoter. It is likely to be the first gene therapy to be approved as, in June 2022, EMA recommended granting conditional marketing authorization in the EU. The expression cassette is inserted between two AAV serotype 2 inverted terminal repeats [69]. The non-enveloped icosahedral AAV5 capsid delivers the transgene predominantly to the liver [69]. The vector itself is manufactured using a baculovirus–*Spodoptera frugiperda* (Sf9) insect–cell production system [69].

In an ongoing phase ½ study (NCT02576795), an infusion of valoctocogene roxaparvovec was administered to 15 adults with severe hemophilia A (FVIII ≤ 1 IU/dL) at doses of 6 × 10^12^ viral genome (vg)/kg (n = 1), 2 × 10^13^ vg/kg (n = 1), 6 × 10^13^ vg/kg (n = 7) or 4 × 10^13^ vg/kg (n = 6) [64,65,69]. Four of seven and three of six participants in the 6 × 10^13^ vg/kg and 4 × 10^13^ vg/kg cohorts, respectively, maintained median FVIII levels > 5 IU/dL at 4 and 5 years after administration [64]. Individuals in the 6 × 10^13^ vg/kg cohort had a mean ABR of 0.7 treated bleeds/year during year 5, resulting in a cumulative mean ABR of 0.8 treated bleeds/year from week 5 onward (95% reduction from baseline) [64]. Individuals in the 4 × 10^13^ vg/kg cohort had a mean ABR of 1.7 treated bleeds/year during year 4, resulting in a cumulative mean ABR of 1.0 treated bleeds/year from week 5 onward, a 92% reduction from baseline [64]. QoL was maintained in patients receiving the higher dose [64]. The most common adverse events were transient, asymptomatic, and mild-to-moderate ALT elevations [64].

In the phase 3 study (NCT03370913), a single 6 × 10^13^ vg/kg valoctocogene roxaparvovec infusion was administered to 134 adult males with severe hemophilia A (FVIII ≤ 1 IU/dL) on FVIII prophylaxis (negative for FVIII inhibitors) [63]. CSA-assessed FVIII activity increased by a mean of 41.9 IU/dL at weeks 49–52 (baseline FVIII activity was assumed to be 1 IU/dL, as there was no washout of FVIII therapy before valoctocogene roxaparvovec infusion). An 83.8% reduction in mean ABR and 98.6% reduction in FVIII infusion rate were reported. Data for two years’ post-infusion are available for 17 study participants [63]. In these individuals, the mean FVIII activity level was 24.4 IU/dL at week 104, compared with 42.2 IU/dL at weeks 49–52 after infusion. At week 104, 18% (n = 3) of study participants had a median FVIII activity level of ≥40 IU/dL, 59% (n = 10) had an activity level of >5 to <40 IU/dL, and 24% (n = 4) had an activity level of <5 IU/dL. All study participants experienced at least one adverse event, with an increase in ALT (85.8% of participants) and aspartate aminotransferase (AST; 35.1%) levels, headache (38.1%) and nausea (37.3%) being the most common. Most events were of grade 1 or 2, but 8.2% of patients had a grade 3 increase in ALT, and five patients reported a serious adverse event that was considered to be treatment-related. All serious adverse events resolved, as did 96.2% of ALT elevations (with treatment), and no participants died, withdrew from the study because of adverse events, or developed FVIII inhibitors. FVIII activity levels >150 IU/dL were found in 7 (5.2%) patients at weeks 49–52, but none developed thromboembolism [63].

### 4.2. Dirloctocogene Samoparvovec

Dirloctocogene samoparvovec (SPK-8011) is a recombinant-AAV vector consisting of SPK200 (a bioengineered capsid derived from AAV3 (specifically, subtype LK03)) with a liver-specific, truncated transthyretin enhancer and promoter, a synthetic intron sequence, and codon-optimized FVIII cDNA encoding FVIII-SQ and manufactured with transient triple transfection of human embryonic kidney cells (HEK293 cells) [58].

In a phase ½ trial (NCT03003533/NCT03432520), 18 men with hemophilia A were enrolled in four dose cohorts; the lowest-dose cohort received 5 × 10^11^ vg/kg and the highest-dose cohort received 2 × 10^12^ vg/kg, with glucocorticoids administered in cases of suspected immune response [58]. Two participants lost all FVIII expression because of an anti-AAV capsid cellular immune response that was not sensitive to immune suppression. In the remaining 16 men, FVIII expression was maintained, with 12 men followed for >2 years. These men had a 91.5% reduction in ABR after dirloctocogene samoparvovec administration. Elevated ALT levels were seen in seven study participants, the majority of which were mild, with the exception of a grade 2 elevation in one participant that required hospitalization for treatment. Adverse events related to glucocorticoids were reported in four participants. No cases of FVIII inhibitory antibody development were reported.

### 4.3. SPK-8016

A phase ½, open-label, non-randomized, dose-finding study is investigating the efficacy of SPK-8016 in adult men with clinically severe hemophilia A who have not developed FVIII inhibitors and had no AAV neutralizing antibodies (NCT03734588) [70]. In three patients, there was evidence for unstable FVIII:C and cellular immunity against AAV. Patients were put on daily oral corticosteroids at weeks 3–7 for 43–38 weeks. Preliminary results demonstrated sustained FVIII levels (6.2–21.8%) after 52 weeks in four men who received SPK-8016 at a dose of 5 × 10^11^ vg/kg. An optimization of the immunomodulatory regimen is being developed with the potential for obtaining clinically meaningful FVIII activity using low vector doses [70].

### 4.4. Giroctocogene Fitelparvovec

Giroctocogene fitelparvovec comprises an AAV serotype 6 vector (AAV2/6) encoding the cDNA for B domain-deleted human FVIII. This gene therapy has been investigated in a phase ½ trial (NCT03061201) in which 11 patients were each given four ascending doses: 9 × 10^11^, 2 × 10^12^, 1 × 10^13^, and 3 × 10^13^ vg/kg (n = 2, 2, 2 and 5 patients, respectively) [71]. Four patients in the 3 × 10^13^ vg/kg cohort have available data, which demonstrated mean FVIII activity maintained in the mild to normal range (30.9% measured by CCA; 46.4% measured by one-stage assay) to week 104. The ABR was zero for the first-year post-infusion and 0.9 throughout the follow-up, and two patients experienced a total of three bleeding events (two traumatic, one unknown; one in a target joint) that necessitated treatment with exogenous FVIII. No patients have resumed prophylaxis. Common treatment-related adverse events were elevated liver enzyme levels (ALT n = 5/11 patients, and AST n = 3/11), pyrexia (n = 3/11), and tachycardia (n = 2/11). Four patients in the 3 × 10^13^ vg/kg cohort had an ALT elevation that required >7 days of corticosteroid treatment, but these were managed with a tapering course of corticosteroids, and FVIII activity was maintained. No patients developed inhibitors to FVIII.

Giroctocogene fitelparvovec was being evaluated in a phase 3 study in men with moderately severe to severe hemophilia A (AFFINE; NCT04370054) [57], but the US Food and Drug Administration has placed this study on hold until proposed protocol amendments have been implemented, due to FVIII levels being >150% in some participants [57].

### 4.5. TAK-754

A phase ½ trial (NCT03370172) is currently evaluating TAK-754, a modified AAV serotype 8 gene therapy, in four men with severe hemophilia A [56]. Participants were treated in two ascending dose cohorts (2 × 10^12^ capsid particles [cp]/kg and 6 × 10^12^ cp/kg) [56]. FVIII expression declined significantly during the tapering of corticosteroids, and three of the four patients have resumed FVIII replacement therapy after ≥10 months’ follow-up. The utilization of corticosteroids did not prevent the loss of transgene expression. The reason behind transcript expression loss is being investigated [56].

### 4.6. BAY 2599023

BAY 2599023 (AAVhu37.hFVIIIco) comprises an AAV vector with a capsid serotype hu37 (AAVhu37), and a genome that directs expression of a codon-optimized B-domain-deleted human FVIII under the control of a liver-specific promoter/enhancer combination [67].

A phase ½ study (NCT03588299) is being conducted in adult males who have severe hemophilia A with no history of FVIII inhibitors, no detectable immunity to the AAVhu37 capsid, and ≥150 exposure days to FVIII products [67,68]. Nine patients have received a single infusion of BAY 2599023 at doses of 0.5, 1.0, and 2.0 × 10^13^ gene copies/kg (n = 2, 2, and 5, respectively) [67,68]. Successful proof-of-concept was achieved with measurable, stable expression of endogenous FVIII for up to >23 months [67,68]. Bleeding protection has been observed, with patients in the two higher dose groups not requiring prophylaxis with FVIII products from approximately 6–12 weeks after gene transfer, and no spontaneous bleeds requiring treatment being reported once FVIII levels reached >11 IU/dL. Mild-to-moderate ALT elevations have been observed in five patients but these were successfully managed with corticosteroids or famotidine [68].

### 4.7. AAV8-HLP-hFVIII-V3

AAV-HLP-hFVIII-V3 encodes a 17-amino-acid peptide with six N-linked glycosylation motifs from the human FVIII B domain, pseudotyped with AAV serotype 8 capsid, and has been shown to mediate three-fold higher FVIII expression compared with valoctocogene roxaparvovec in an animal model [61]. In a phase ½ study (NCT03001830), three men with severe hemophilia A received a single infusion of AAV8-HLP-hFVIII-V3 [61], with one man receiving a dose of 6 × 10^11^ vg/kg and the subsequent two patients each receiving a dose of 2 × 10^12^ vg/kg. The patients were followed up for 13–47 weeks after vector administration. FVIII activity levels were >5% in all three men, with normalization of FVIII levels in one patient. Serum ALT levels were elevated in two of the patients between 4 and 6 weeks after gene transfer but this responded to corticosteroids, with no loss of transgene expression. No study participants have developed FVIII inhibitors.

## 5. Opportunities Associated with Gene Therapy in Hemophilia A

Gene therapy is a disease-transforming therapy that has the potential to become the new standard for the treatment of patients with hemophilia A [72]. It may enable the patient to achieve a sustained physiological level of endogenously produced FVIII protein that could provide effective prophylaxis without the need for exogenous factor replacement therapy [40,42]. Such continuous endogenous expression of physiological levels of FVIII can be expected to eliminate breakthrough bleeding and micro-hemorrhages [72]. Gene therapy may, thus, provide protection against further bleeding-induced joint damage [73]. While the clinical studies reported to date indicate a cessation of joint bleeds [63,65], further data are needed to further investigate whether this leads to a reduction in joint damage, although this can be anticipated.

Given that even minimal increases in FVIII levels can lead to significantly improved bleeding outcomes, it is expected that gene therapy will provide a resultant improvement in the QoL of the individual with hemophilia A [38,72].

## 6. Challenges of Gene Therapy in Hemophilia A

Successful gene therapy was first reported, in 2011, with intravenously administered AAV-based liver-directed gene therapy in patients with severe hemophilia B [74]. In contrast, gene therapy directed at hemophilia A has lagged behind that directed at hemophilia B, largely due to the limitations inherent in the size of the FVIII gene and the structure of FVIII, problems achieving therapeutic levels of the transgenic protein, and cellular immune responses to the capsid of the AAV vector [75] (also identified in trials of therapy for hemophilia B, although to a lesser extent), as well as issues related to the duration and variability of this form of therapy [1,4,6].

### 6.1. FVIII Structure

The gene for FVIII is located at the tip of the long arm of the X chromosome. At over 180 kb, it is one of the largest known genes. It comprises 26 exons, which encode a polypeptide chain of 2351 amino acids (19 signal peptides and a 2332-amino-acid mature polypeptide chain). Factor VIII is composed of six domains, A1-A2-B-A3-C1-C2, with three acidic regions (α1-3) bordering the A domains. The A domains are each approximately 330 residues in length, the B domain approximately 900 residues, and the C domains each approximately 150 residues [76].

FVIII, and the FVIII complementary DNA (cDNA) for FVIII (~7 kb), exceed the packaging capacity of AAV vectors (~5 kb) [40]. This restriction has been overcome by using FVIII transgenes optimized for packaging through the removal of the B domain, which comprises a large proportion of the gene but is not required for clotting function [48,77,78,79]. All current FVIII transgene constructs utilize a B domain-deleted (or truncated) cDNA [1,80].

### 6.2. FVIII Expression

The target cell for FVIII gene therapy is the hepatocyte, which is not the native cell for the endogenous FVIII synthesis [7,8]. Nevertheless, expression of FVIII in hepatocytes generates a functional and physiological protein that has restored hemostasis in animal models and clinical studies [81,82]. In contrast, other currently available therapies involve the administration of exogenous FVIII by infusion or the subcutaneous administration of an FVIII-mimicking compound [2,14].

The expression of FVIII and the duration of this expression between patients is variable, not only between the different studies, but also within dose cohorts and within the patients themselves over time [83]. Low levels of FVIII expression may be overcome by introducing liver-specific promoters and enhancers [84].

### 6.3. Infusion-Related Adverse Reactions

Infusion-related adverse reactions can occur with gene therapy for hemophilia A, but are generally infrequent, resolve with treatment and/or slowing or pausing the infusion, and do not usually prevent completion of the infusion. In a phase ½ study (NCT02576795) of valoctocogene roxaparvovec (an AAV5-based gene therapy encoding human B domain-deleted FVIII), one patient was hospitalized due to grade 2 pyrexia with myalgia and headache that occurred within 24 h after the infusion; the symptoms resolved within 48 h after treatment with acetaminophen (paracetamol) [65]. In a phase 3 study of valoctocogene roxaparvovec (NCT03370913), seven study participants (5.2%) had systemic hypersensitivity and three reported serious infusion-related reactions (maculopapular rash and presyncope; anaphylactic reaction; and hypersensitivity reaction) during or shortly after the infusion [63]. However, the infusion reactions were managed by slowing or pausing the infusion and administering supportive medications (e.g., antihistamines, antipyretics, or glucocorticoids), such that all participants completed the infusion. During a phase ½ trial of dirloctocogene samoparvovec (NCT03003533; NCT03432520), one participant had an acute infusion-related reaction, characterized by vomiting, myalgia, back pain, and pyrexia 12 h after administration, which resolved within 72 h after antipyretic treatment [58]. One patient experienced hypotension and fever approximately 6 h after infusion of the highest dose of giroctocogene fitelparvovec in a phase ½ study (NCT03061201); the events fully resolved with treatment and did not delay post-infusion discharge [60].

### 6.4. Anti-AAV Neutralizing Antibodies

Exposure to wild-type AAV results in priming of the body’s immune system against the virus, with the development of both humoral and T-cell immunity that can neutralize the vector and reduce treatment efficacy [85,86]. At present, patient selection in gene therapy trials in hemophilia A includes screening for pre-existing immunity to identify and enroll patients who are negative for anti-AAV capsid neutralizing antibodies [6,87].

The prevalence of anti-AAV immunoglobulin G and neutralizing antibodies differs for each AAV type within the general population. Although data are somewhat inconsistent, studies suggest a higher prevalence of antibodies against AAV1 and AAV2 than against AAV5, AAV6, AAV8, and AAV9 [88,89,90,91]. This suggests that the use of vectors based on the latter AAV types may avoid pre-existing immunity and, therefore, have advantages for use in gene therapy.

Individuals without pre-existing immunity who are treated with AAV-based gene therapy are likely to develop high-titer antibodies to the vector. For example, in a phase ½ study of valoctocogene roxaparvovec in which patients with pre-existing immunity to AAV5 were excluded from enrolment, anti-AAV5 antibodies were detected in all patients (n = 15) after treatment (follow-up was at least 104 weeks, with a maximum of 183 weeks) [92]. All patients had seroconverted at week 8 post-treatment (the first time point assessed). The titer peaked 40 weeks after administration and remained stable through to week 104, with no apparent dose–response relationship. Further, there was no correlation between the immune response and safety (alanine transaminase (ALT) levels) or efficacy measures. Cross-reactivity to other AVV serotypes was also observed [92].

Further research is needed to determine the implications of such immune responses for the repeated administration of gene therapy, whether repeated administration of the same gene therapy or that of different therapies that use AAV-type vectors for which cross-reactivity has been observed.

### 6.5. Elevation of Liver Enzymes

AAV-based gene therapy may induce a degree of hepatotoxicity, which is usually transient and asymptomatic. Increased levels of serum ALT are commonly seen. The mechanism of this toxicity has not been clearly established but could be related to the development of an anti-AAV capsid peptide cytotoxic T cell response, endoplasmic reticulum stress, and associated hepatocyte apoptosis due to high expression of FVIII, or a direct effect of vector particle load [80].

Clinical studies in patients with hemophilia A have reported increases in ALT level in a high proportion of patients [56,58,60,61,63,65,67,69,70]. In a phase ½ study of valoctocogene roxaparvovec in patients with hemophilia A, an increase in ALT was reported in 11 of the 15 study participants (73.3%; 14 events). The increases were not generally associated with loss of FVIII activity or a T-cell immune response to viral capsid peptides [65]. All elevations in ALT were mild (13 events grade 1 and 1 event grade 2), non-serious and transient, and there were no symptoms or sequelae suggestive of clinically significant hepatocyte injury or liver dysfunction [65]. There were no consistent cellular immune responses to FVIII or AAV5 capsid detected in any participant [65]. Similarly, transient elevations of ALT were seen in patients participating in the phase 3 study of valoctocogene roxaparvovec [63]. The elevations occurred in 115 participants (85.8%) and lasted for a median duration of 15 days. While most events were grade 1 or 2 in severity, 11 participants (8.2%) experienced 12 events of grade 3 ALT elevations (>5 to 20 times the upper limit of normal), with two events deemed to be serious, although none met the criteria for significant liver injury. All events were managed with glucocorticoids and resolved [63]. In a phase ½ study of dirloctocogene samoparvovec, elevated ALT levels were seen in seven participants (39%; 13 events) [58]. All the elevations were considered mild, except for that in one patient who experienced a grade 2 elevation that constituted a serious AE.

During clinical studies, increases in ALT levels were managed with a tapering course of glucocorticoids [58,65,69]. In the phase 3 study of valoctocogene roxaparvovec, 79.1% of patients received glucocorticoids to manage ALT level elevations, with a median duration of treatment of 230 days (range 22–551 days) [63].

Participants receiving prednisolone also showed a concomitant increase in FVIII activity after valoctocogene roxaparvovec [69], raising the question of whether prednisolone may directly regulate transgene expression; however, in animal models of hemophilia treated with valoctocogene roxaparvovec, prednisolone did not regulate FVIII expression [93].

While steroids may provide immunosuppression, they can be associated with adverse effects (e.g., weight gain, edema, irritability, adrenal insufficiency, negative effects on bone health, diabetes, and hypertension), as well as an increased risk of infection [35,58]. Indeed, almost 72% of the patients treated with glucocorticoids in the phase 3 study of valoctocogene roxaparvovec experienced such adverse events, with the most common being acne, insomnia, Cushing’s syndrome, and weight gain [63]. Serious adverse events attributed to glucocorticoids occurred in 2.7% of study participants [63]. In a few cases, hepatotoxicity may be unresponsive to oral steroids, requiring the administration of intravenous methylprednisolone or alternative immune-regulatory agents (e.g., tacrolimus or azathioprine) [80].

### 6.6. Oncogenesis

The risk of genomic insertional mutagenesis after AAV-mediated gene transfer is considered to be low due to the episomic nature of the transinfected cDNA [6]. Durable FVIII expression has been observed in an animal model of severe hemophilia A, with no late-toxicity or oncogenesis [94]. A study in hemophilia dogs treated with AAV8 or AAV9 vectors expressing canine FVIII and followed for 10 years did not find any evidence of hepatic tumors or altered liver function, although there were some AAV integration events in genomic DNA and clonal expansion of cells that harbored vector integration in genes potentially associated with growth control [54].

A patient enrolled in a phase 3 clinical trial of a gene therapy for hemophilia B (etranacogene dezaparvovec) developed a hepatocellular carcinoma, although an independent investigation concluded that it was ‘highly unlikely’ that this was caused by the therapy, given the low level of vector integration in the patient’s tissue and the finding that the patient had a number of risk factors for hepatocellular carcinoma [95]. There is currently no evidence that gene therapies for hemophilia A are associated with a risk of oncogenesis. A patient who received valoctocogene roxaparvovec in a phase ½ trial developed salivary gland cancer, but it was deemed unrelated to the therapy [96]. There was no evidence of hepatocellular carcinoma in a phase ½ trial of dirloctocogene samoparvovec; this was specifically reported, having been investigated using ultrasonographic assessment of the liver [58]. In a phase ½ trial of giroctocogene fitelparvovec, no ‘hepatic masses’ were detected [71].

Ongoing investigation in animal models, long-term follow-up of clinical studies, and surveillance using long-term registries of patients treated with gene therapy are required to confirm that the risk of oncogenesis is minimal.

### 6.7. Durability and Variability of Transgene Expression

The clinical trials completed so far have not answered fully all issues related to gene therapy in hemophilia A. Open questions remain as to why expression levels of FVIII decrease over time and why there is intra- and inter-patient variability [83]. It is yet to be determined whether the vector serotype, manufacturing process, expression of FVIII in hepatocytes, FVIII protein unfolding, episomal structural changes, or other factors contribute to this decline/variability in FVIII levels [1]. The impact of liver growth and hepatocyte turnover on AAV vector genome persistence, and the efficacy and durability of factor expression in adults, adolescents, and pediatric groups is also still unclear [97]. Variability may or may not be related to durability [98]. Identifying the causes of inter- and intra-individual variability needs to be a research priority, as finding the root causes of the variability might permit potential mitigants to be identified [98].

In light of these questions, the durability/variability of hepatocyte-expressed FVIII in humans continues to be investigated in clinical trials [1,6]. In a phase ½ study in 15 patients with hemophilia A who received a single dose of valoctocogene roxaparvovec, follow-up data to 5 years indicated that 10 out of 13 participants continued to have FVIII levels above the severe hemophilia A range (>1 IU/dL) [64]. Data from patients up to 2 years post-infusion who are taking part in the phase 3 study of valoctocogene roxaparvovec (n = 17) are also encouraging, with 76% having a median FVIII activity level of >5 IU/dL at this time point, and 18% having a level of ≥40 IU/dL [63]. In a phase ½ study of dirloctocogene samoparvovec, FVIII expression was maintained in 16/18 individuals, with the mean FVIII activity being 6.9% of the normal value in an FVIII chromogenic assay (CSA) at >52 weeks after vector administration [99]. However, two of 18 individuals with hemophilia A lost all FVIII expression because of an anti-AAV capsid cellular immune response that was not sensitive to immune suppression [99].

Unfortunately, the positive sustained responses in the majority of patients in clinical trials poses concerns that these patients may be lost to follow-up [100]. Long-term surveillance of these patients is essential with regards to their clinical bleeding patterns, levels of transgenic clotting factor, the possible development of clotting factor immune response, and the need for regular hepatic imaging assessment to ensure that no neoplastic process is developing [100]. There is also a need for long-term follow-up and post-marketing surveillance through registries to establish if the efficacy and tolerability of gene therapy observed in clinical trials are maintained long term in trial participants and in the real-world population [97].

### 6.8. Patient Expectations

Gene therapy has the potential to change the quality of life of patients with hemophilia A by reducing the frequency of bleeding events, thereby allowing patients to lead lives that are less impacted by the disease [83,101,102]. However, it is important that patient expectations surrounding their eligibility, access to treatment, and treatment outcomes are managed appropriately, as this therapy may not be suitable for all patients [83,102,103]. This is because there is variability in FVIII expression between patients, FVIII expression may not be durable in all patients, and some patients may be positive for anti-AAV capsid neutralizing antibodies and, therefore, not suitable candidates for gene therapy.

People living with hemophilia A need to be fully informed regarding the potential benefits and risks of gene therapy [102]. It will be essential that physicians pay close attention to what information they share with their patients, choose the optimum way to do so, and determine how to ensure sufficient patient understanding of the information provided [102].

At present, there are no data on the effects of gene therapy in children with hemophilia, and no ongoing trials. As such, the expectations of the parents of these children regarding the appropriateness of gene therapy in younger patients with hemophilia will need to be managed accordingly.

## 7. Assessment of Efficacy of FVIII Expression

The measurement of FVIII activity is a direct and accessible way to determine transgene expression, but there are differences in standard clinical laboratory measurements of clotting activity, with the one-stage assay providing higher levels of FVIII activity than the CSA [104,105]. One-stage assays and/or chromogenic assays have been employed to measure circulating FVIII or FIX levels and duration of transgene expression in liver-directed gene therapy studies. A 1.6-fold difference between the values obtained by the two methods was encountered in comparative studies for the B-domain-deleted FVIII-SQ in valoctocogene roxaparvovec AAV5-FVIII-SQ [105]. Likewise, differences between chromogenic and one-stage FIX assays with one-stage values ≅ 2-fold higher than chromogenic assay values were found for the transgene expression of Factor IX–R338L [106]. In our opinion, clinical outcomes are likely to define which assay best measures FVIII/FIX levels, thus uncovering the real efficacy/safety profile of the transgenic factors. In the only phase 3 trial of gene therapy for hemophilia completed and published to date (of valoctocogene roxaparvovec), the primary endpoint was the change from baseline in median FVIII activity during weeks 49–52, assessed using the CSA [62,63]. We would like to point out that, as successfully experimented with the BDD-FVIII recombinant product, the use of a standard reference factor provided by the company, would assure accuracy and precision in measuring the achieved levels of a transgenic factor in treated patients in a simple one-stage assay [107].

In clinical trials, a holistic assessment of patient outcomes is needed [80,83]. Measures of QoL and hemophilia-specific patient-reported outcomes need to be used [83,101]. Annualized bleeding rate (ABR) is a patient-reported outcome in which the occurrence of bleeding events, their location, severity and whether there was a precipitating event is recorded by the patient. The collection of QoL outcomes will enable a better understanding of the cost–benefit of gene therapies in patients with hemophilia A [1]. An early cost-effectiveness analysis compared valoctocogene roxaparvovec with standard half-life FVIII or emicizumab in patients with severe hemophilia A without FVIII antibodies using a Dutch societal perspective with a 10-year time horizon [108]. This suggested that valoctocogene roxaparvovec may result in improved health and lower cost compared with prophylactic FVIII and emicizumab.

One issue that remains to be elucidated with any gene therapy for hemophilia A is whether this treatment will longer-term benefit joint health and structure, as determined by imaging analysis [80].

## 8. Implementing Gene Therapy in a Real-World Setting

Gene therapy is a complex treatment process and when approved for reimbursement it will likely first be offered at comprehensive care centers with significant expertise in the management of patients with hemophilia. A recent joint publication from the European Association for Haemophilia and Allied Disorders and the European Haemophilia Consortium suggested that a modified ‘hub-and-spoke’ model, incorporating a long-term safety and efficacy surveillance system, be introduced to ensure appropriate prescription, administration, and monitoring of gene therapy in patients with severe hemophilia A [109,110].

Given the imminent approval of gene therapy for hemophilia A in Italy, there is a need to determine how this treatment service will be organized on national and local levels and how patients who are candidates for gene therapy in Italy will be assessed, treated, and managed [111]. In our opinion, the hub-and-spoke model should be followed for the best cost-effectiveness and resource utilization. Hubs should manage all aspects of therapy delivery and data collection, while spokes should take care of pre-screening and patient selection. A set of detailed selection criteria will need to be elaborated by multidisciplinary teams present in the centers involved; however, there will have to be flexibility in the system as patients’ individual situations can be very different and should not lead to an a priori exclusion. We believe that contacts with patients should be kept by a single designated member of the multidisciplinary team, who will act as a point of contact throughout the procedure and the follow-up. Such a person will have the preparation to provide information to the patient including the possibility of transgene expression loss and the necessity of returning to other treatments. An active engagement with patients is very important and can be obtained through an adequate explanation and support given to the patient. Patient association groups can play an important role in this aspect. Adequate laboratory surveillance needs to be available at all stages pre- and post-procedure. In particular, a reliable standardized determination of anti-AAV antibodies and monitoring of FVIII levels must be ensured together with safety monitoring (e.g., liver function). A structured follow-up must be provided to all patients, especially, in the first year after gene therapy.

## 9. Conclusions

The current treatment of hemophilia A involves frequent intravenous injections and the risk of FVIII inhibitor development, which can negatively impact patient QoL. Gene therapy for patients with hemophilia A represents the potential for a single treatment that could allow for long-term expression of the deficient FVIII, with the maintenance of steady-state plasma FVIII concentrations, thereby minimizing bleeding episodes for the whole lifetime of the recipient and decreasing the burden of their disease. FVIII expressed through gene therapy is a physiological protein rather than a mimicking factor or an infused exogenous FVIII concentrate, and steady-state physiological plasma FVIII levels may avoid the deterioration of joint status.

Gene therapy for the management of hemophilia A has now moved beyond proof of concept, with the realistic expectation that it may become available to patients in European countries, including Italy, in 2022 [49,80]. It is likely that the first licensed product will be valoctocogene roxaparvovec, for which the longest duration efficacy and safety data (5 years) for any gene therapy currently under investigation are available. These data indicate sustained secretion of FVIII in patients who have received valoctocogene roxaparvovec, such that ABR and FVIII replacement therapy were minimized [64].

We believe that improved patient selection and management of patient expectations will enable the benefits of gene therapy to be maximized. Consequently, gene therapy could represent a paradigm shift, becoming a new reference standard in the treatment of patients with hemophilia A, enabling long-lasting treatment and improved clinical and patient-centered outcomes, including enhanced QoL, for many patients with this disease.

## Figures and Tables

**Table 1:** Gene therapy trials involving adeno-associated viral (AAV) vectors in patients with hemophilia A.

Gene Therapy	Adenovirus Serotype	Clinical Stage (Trial Name; ClinicalTrials.gov Identifier)	Starting Year/Status on ClinicalTrials.gov	Company
Valoctocogene roxaparvovec (AAV5-hFVIII-SQ; BMN 270)	AAV5	Phase 3 (GENEr8-1; NCT03370913; NCT03392974) [63]	2017, active, not recruiting; 2018, active, not recruiting	BioMarin
		Phase ½ (NCT02576795) [64,65,69]	2015, active, not recruiting	
Dirloctocogene samoparvovec (SPK 8011)	AAV3	Phase ½ (NCT03003533; NCT03432520) [58]	2016, recruiting; 2018, enrolling by invitation	Spark Therapeutics; Roche
SPK-8016	AAV	Phase ½ (NCT03734588) [70]	2018, recruiting	Spark Therapeutics; Roche
Giroctocogene fitelparvovec (SB-525; PF-07055480)	rAAV6	Phase 3 (AFFINE; NCT04370054) [57] [on hold]	2020, active, not recruiting	Pfizer (Sangamo)
		Phase ½ (NCT03061201) [60,71]	2017, active, not recruiting	
TAK-754 (previously SHP654 and BAX 888)	AAV8	Phase ½ (NCT03370172) [56]	2017, active, not recruiting	Takeda-Shire
BAY 2599023	AAVhu37	Phase ½ (NCT03588299) [67]	2018, recruiting	Bayer-Ultragenix Pharmaceutics
AAV8-HLP-hFVIII-V3	AAV8	Phase ½ (GO-8; NCT03001830) [61]	2016, recruiting	University College, London

## Data Availability

No new data were created or analyzed in this study. Data sharing is not applicable to this article.
